# Supplementary material for: The economic burden of dengue: a systematic literature review of unit costs for non-fatal episodes treated in the formal healthcare system
Source: BMC Infect Dis. 2026 Jan 16;26:320. doi: 10.1186/s12879-025-12451-8 (PMC12896024; doi:10.1186/s12879-025-12451-8)
Supplement: Supplementary file 1 — Supplementary Material 1 [file 12879_2025_12451_MOESM2_ESM.docx]

**Appendix 1 PRISMA 2020 checklist**

| **Section and Topic** | **Item #** | **Checklist item** | **Location/page where item is reported** |
| --- | --- | --- | --- |
| **TITLE** | | |  |
| Title | 1 | Identify the report as a systematic review. | 1 |
| **ABSTRACT** | | |  |
| Abstract | 2 | See the PRISMA 2020 for Abstracts checklist. | 2 |
| **INTRODUCTION** | | |  |
| Rationale | 3 | Describe the rationale for the review in the context of existing knowledge. | 4 |
| Objectives | 4 | Provide an explicit statement of the objective(s) or question(s) the review addresses. | 4 |
| **METHODS** | | |  |
| Eligibility criteria | 5 | Specify the inclusion and exclusion criteria for the review and how studies were grouped for the syntheses. | 4-5 |
| Information sources | 6 | Specify all databases, registers, websites, organisations, reference lists and other sources searched or consulted to identify studies. Specify the date when each source was last searched or consulted. | 4-5 |
| Search strategy | 7 | Present the full search strategies for all databases, registers and websites, including any filters and limits used. | 4-5, Appendix 2 |
| Selection process | 8 | Specify the methods used to decide whether a study met the inclusion criteria of the review, including how many reviewers screened each record and each report retrieved, whether they worked independently, and if applicable, details of automation tools used in the process. | 4-5,  Appendix 2 |
| Data collection process | 9 | Specify the methods used to collect data from reports, including how many reviewers collected data from each report, whether they worked independently, any processes for obtaining or confirming data from study investigators, and if applicable, details of automation tools used in the process. | 5-7 |
| Data items | 10a | List and define all outcomes for which data were sought. Specify whether all results that were compatible with each outcome domain in each study were sought (e.g. for all measures, time points, analyses), and if not, the methods used to decide which results to collect. | 5 |
|  | 10b | List and define all other variables for which data were sought (e.g. participant and intervention characteristics, funding sources). Describe any assumptions made about any missing or unclear information. | 5 |
| Study risk of bias assessment | 11 | Specify the methods used to assess risk of bias in the included studies, including details of the tool(s) used, how many reviewers assessed each study and whether they worked independently, and if applicable, details of automation tools used in the process. | 5 |
| Effect measures | 12 | Specify for each outcome the effect measure(s) (e.g. risk ratio, mean difference) used in the synthesis or presentation of results. | 5 |
| Synthesis methods | 13a | Describe the processes used to decide which studies were eligible for each synthesis (e.g. tabulating the study intervention characteristics and comparing against the planned groups for each synthesis (item #5)). | 6-7 |
|  | 13b | Describe any methods required to prepare the data for presentation or synthesis, such as handling of missing summary statistics, or data conversions. | 6-7 |
|  | 13c | Describe any methods used to tabulate or visually display results of individual studies and syntheses. | 6-7 |
|  | 13d | Describe any methods used to synthesize results and provide a rationale for the choice(s). If meta-analysis was performed, describe the model(s), method(s) to identify the presence and extent of statistical heterogeneity, and software package(s) used. | 6-7 |
|  | 13e | Describe any methods used to explore possible causes of heterogeneity among study results (e.g. subgroup analysis, meta-regression). | 6-7 |
|  | 13f | Describe any sensitivity analyses conducted to assess robustness of the synthesized results. | No |
| Reporting bias assessment | 14 | Describe any methods used to assess risk of bias due to missing results in a synthesis (arising from reporting biases). | No |
| Certainty assessment | 15 | Describe any methods used to assess certainty (or confidence) in the body of evidence for an outcome. | No |
| **RESULTS** | | |  |
| Study selection | 16a | Describe the results of the search and selection process, from the number of records identified in the search to the number of studies included in the review, ideally using a flow diagram. | 8-10 |
|  | 16b | Cite studies that might appear to meet the inclusion criteria, but which were excluded, and explain why they were excluded. | No |
| Study characteristics | 17 | Cite each included study and present its characteristics. | 9-10 |
| Risk of bias in studies | 18 | Present assessments of risk of bias for each included study. | No |
| Results of individual studies | 19 | For all outcomes, present, for each study: (a) summary statistics for each group (where appropriate) and (b) an effect estimate and its precision (e.g. confidence/credible interval), ideally using structured tables or plots. | 8-10 Appendix 5 |
| Results of syntheses | 20a | For each synthesis, briefly summarise the characteristics and risk of bias among contributing studies. | 10-16 |
|  | 20b | Present results of all statistical syntheses conducted. If meta-analysis was done, present for each the summary estimate and its precision (e.g. confidence/credible interval) and measures of statistical heterogeneity. If comparing groups, describe the direction of the effect. | 10-16 |
|  | 20c | Present results of all investigations of possible causes of heterogeneity among study results. | 9-10, 13-15 |
|  | 20d | Present results of all sensitivity analyses conducted to assess the robustness of the synthesized results. | No |
| Reporting biases | 21 | Present assessments of risk of bias due to missing results (arising from reporting biases) for each synthesis assessed. | No |
| Certainty of evidence | 22 | Present assessments of certainty (or confidence) in the body of evidence for each outcome assessed. | No |
| **DISCUSSION** | | |  |
| Discussion | 23a | Provide a general interpretation of the results in the context of other evidence. | 16-20 |
|  | 23b | Discuss any limitations of the evidence included in the review. | 20-21 |
|  | 23c | Discuss any limitations of the review processes used. | 20-21 |
|  | 23d | Discuss implications of the results for practice, policy, and future research. | 22 |
| **OTHER INFORMATION** | | |  |
| Registration and protocol | 24a | Provide registration information for the review, including register name and registration number, or state that the review was not registered. | No |
|  | 24b | Indicate where the review protocol can be accessed, or state that a protocol was not prepared. | No |
|  | 24c | Describe and explain any amendments to information provided at registration or in the protocol. | No |
| Support | 25 | Describe sources of financial or non-financial support for the review, and the role of the funders or sponsors in the review. | No |
| Competing interests | 26 | Declare any competing interests of review authors. | No |
| Availability of data, code and other materials | 27 | Report which of the following are publicly available and where they can be found: template data collection forms; data extracted from included studies; data used for all analyses; analytic code; any other materials used in the review. | Fig 1, Appendix 2, Appendix 5 |

**Reference:** (1)

**Appendix 2 Details of keyword used in each database.**

| **Database** | **Full keywords** |
| --- | --- |
| MEDLINE via Ovid &  EMBASE via Ovid | 1 exp Dengue/ or exp Severe Dengue/ or exp Dengue Virus/  2 dengue.mp. [mp=title, book title, abstract, original title, name of substance word, subject heading word, floating sub-heading word, keyword heading word, organism supplementary concept word, protocol supplementary concept word, rare disease supplementary concept word, unique identifier, synonyms, population supplementary concept word, anatomy supplementary concept word]  3 DENV.mp. [mp=title, book title, abstract, original title, name of substance word, subject heading word, floating sub-heading word, keyword heading word, organism supplementary concept word, protocol supplementary concept word, rare disease supplementary concept word, unique identifier, synonyms, population supplementary concept word, anatomy supplementary concept word]  4 DENV-1.mp. [mp=title, book title, abstract, original title, name of substance word, subject heading word, floating sub-heading word, keyword heading word, organism supplementary concept word, protocol supplementary concept word, rare disease supplementary concept word, unique identifier, synonyms, population supplementary concept word, anatomy supplementary concept word]  5 DENV-2.mp. [mp=title, book title, abstract, original title, name of substance word, subject heading word, floating sub-heading word, keyword heading word, organism supplementary concept word, protocol supplementary concept word, rare disease supplementary concept word, unique identifier, synonyms, population supplementary concept word, anatomy supplementary concept word]  6 DENV-3.mp. [mp=title, book title, abstract, original title, name of substance word, subject heading word, floating sub-heading word, keyword heading word, organism supplementary concept word, protocol supplementary concept word, rare disease supplementary concept word, unique identifier, synonyms, population supplementary concept word, anatomy supplementary concept word]  7 DENV-4.mp. [mp=title, book title, abstract, original title, name of substance word, subject heading word, floating sub-heading word, keyword heading word, organism supplementary concept word, protocol supplementary concept word, rare disease supplementary concept word, unique identifier, synonyms, population supplementary concept word, anatomy supplementary concept word] 581  8 1 or 2 or 3 or 4 or 5 or 6 or 7  9 exp "Cost of Illness"/ or exp "Costs and Cost Analysis"/  10 exp Financial Stress/  11 exp Health Care Costs/  12 cost of illness.mp. [mp=title, book title, abstract, original title, name of substance word, subject heading word, floating sub-heading word, keyword heading word, organism supplementary concept word, protocol supplementary concept word, rare disease supplementary concept word, unique identifier, synonyms, population supplementary concept word, anatomy supplementary concept word] 34138  13 economic burden.mp. [mp=title, book title, abstract, original title, name of substance word, subject heading word, floating sub-heading word, keyword heading word, organism supplementary concept word, protocol supplementary concept word, rare disease supplementary concept word, unique identifier, synonyms, population supplementary concept word, anatomy supplementary concept word]  14 9 or 10 or 11 or 12 or 13  15 8 and 14 |
| Web of Science | (ALL=(dengue)) AND ALL=(cost of illness OR economic burden) |
| PubMed | (dengue) AND (cost of illness OR economic burden) |

**Appendix 3 Assumptions for data extraction and adjustment.**

For studies adopting a societal perspective that reported direct medical costs borne by the government, direct medical costs borne by households, direct non-medical costs, and productivity costs, the total cost of illness was estimated if not explicitly provided. Thus, our comparison of the total cost of illness included only studies that either reported total costs directly or provided all cost sub-types to ensure consistency.

Similarly, the total direct medical costs were obtained as the sum of the direct medical costs borne by the healthcare provider and those borne by households for studies that reported both. For the studies where it was unclear who paid for the direct medical cost, we assumed it represented the total direct medical cost. Therefore, our comparisons of direct medical costs include only studies that explicitly reported costs borne by both households and the government, as well as studies that reported total direct medical costs without stratification.

If the provider setting was not reported, it was assumed that the data were collected in public hospitals. Furthermore, for studies conducted in the private hospital setting, the cost was assumed to be paid by the household if the study did not report that these costs were borne by private health insurance provider.

It is important to note that any cost data that were ambiguous or uncertain in classification as direct medical, direct non-medical, or productivity costs were excluded from data extraction and subsequent analysis.

**Appendix 4 Assumptions for data analysis.**

Since the World Bank’s exchange rate from Sri Lankan Rupees and Venezuela Bolívar to USD for 2023 were unavailable, the exchange rate from the latest available year was used. Furthermore, for French Guiana, Martinique, Guadeloupe, and Venezuela, where the GDP deflator from the World Bank was not available, the inflation to 2023 values was adjusted using US inflation rates, as this was recommended for countries where GDP deflator data was not available (2). Although WHO classified French Guiana, Martinique, and Guadeloupe as an ‘unknown’ region, we grouped them into the Americas region based on geographic location to facilitate analysis. Finally, in the linear regression analysis, since GDP per capita data for these French territories and Venezuela were not available from the World Bank, we sourced the latest figures from alternative sources. The latest GDP per capita for these French territories were extracted from the Pan America Health Organisation report, while for Venezuela, the GDP per capita in 2023 from the International Monetary Fund was used (3).

**Appendix 5 Details of included studies**

| WHO region | Country | Publication year | Perspective | Provider setting | Treatment setting | Data collection approach | Costing approach | Dengue severity | Age group | Reported total sample size | Study |
| --- | --- | --- | --- | --- | --- | --- | --- | --- | --- | --- | --- |
| African & Western Pacific | Burkina Faso, Kenya, Cambodia | 2019 | Societal | Public | Outpatient, Inpatient | Prospective | Bottom-up | Unclear | Children and adult* | 670 | Lee et al. (4) |
| Americas & Soth-East Asia & Western Pacific | Viet Nam, Thailand, Colombia | 2017 | Societal | Public | Outpatient,  Inpatient | Prospective | Bottom-up | DF and DHF/DSS* | Children, adult | 466 | Lee et al. (5) |
| Americas & Soth-East Asia & Western Pacific | Brazil, El Salvador, Guatemala, Panama, Venezuela, Cambodia, Malaysia, Thailand | 2009 | Societal | Public and private* | Outpatient, Inpatient | Prospective | Macro-costing | DF and DHF/DSS* | Children and adult* | 1,695 | Suaya et al. (6) |
| Americas | Brazil | 2014 | Government & private | Public,  Private | Inpatient and ICU* | Retrospective | Bottom-up | DF and DHF/DSS* | Children and adult* | 288 | Vieira et al. (7) |
| Americas | Brazil | 2015 | Societal | Public, Private | Outpatient, inpatient | Prospective | Micro-costing | DF and DHF/DSS* | Children and adult* | 2,035 | Martelli et al. (8) |
| Americas | Brazil | 2018 | Government | Public | Inpatient | Retrospective | Unclear | DF, DHF/DSS | Children and adult* | 732,195 | Godói et al. (9) |
| Americas | Brazil | 2022 | Private health insurance | Private | Inpatient | Retrospective | Bottom-up | DF and DHF/DSS* | Children and adult* | 64,186 | Abud et al. (10) |
| Americas | Colombia | 2015 | Societal | Public | Outpatient, Inpatient | Retrospective | Bottom-up | DF, DHF/DSS | Children and adult* | 91,742 | Castro Rodriguez et al. (11) |
| Americas | French Guiana, Martinique, Guadeloupe | 2016 | Government | Public | Inpatient and ICU* | Retrospective | Bottom-up | Unclear | Children and adult* | 4,207 | Uhart et al. (12) |
| Americas | Mexico | 2016 | Societal** | Public | Outpatient,  Inpatient,  Intensive care unit | Retrospective | Micro-costing | Unclear | Children and adult* | 2,461 | Zubieta-Zavala et al. (13) |
| Americas | Mexico | 2017 | Household | Public, Private | Outpatient, Inpatient, Informal care | Retrospective | Bottom-up | DF and DHF/DSS* | Children and adult* | 947 | Legorreta-Soberanis et al. (14) |
| Americas | Panama | 2008 | Societal | Public and private* | Outpatient and inpatient* | Prospective | Bottom-up + Macro-costing | DF and DHF/DSS* | Children, adult | 130 | Armien et al. (15) |
| Americas | Peru | 2015 | Household | Public | Outpatient, Inpatient | Retrospective | Bottom-up | DF and DHF/DSS* | Children and adult* | 80 | Salmon-Mulanovich et al. (16) |
| Eastern mediterranean | Pakistan | 2009 | Unclear | Private | Inpatient | Prospective | Bottom-up | DF | Adult | 169 | Riaz et al. (17) |
| Eastern mediterranean | Pakistan | 2011 | Household | Public | Inpatient | Retrospective | Bottom-up | DF and DHF/DSS* | Children and adult* | 250 | Rafique et al. (18) |
| Eastern Mediterranean | Pakistan | 2019 | Household | Public | Inpatient | Prospective | Bottom-up | DF and DHF/DSS* | Children and adult* | Unclear | Jamil et al. (19) |
| Eastern Mediterranean | Saudi Arabia | 2020 | Societal | Public and private* | Outpatient and inpatient* | Retrospective | Bottom-up | DF and DHF/DSS* | Children and adult* | 717 | Akbar et al. (20) |
| South-east Asia | Bangladesh | 2023 | Societal | Public, Private | Inpatient | Retrospective & Prospective | Micro-costing | Unclear | Children and adult* | 302 | Sarker et al. (21) |
| South-east Asia | India | 2008 | Unclear | Private | Inpatient, ICU | Unclear | Bottom-up | DF and DHF/DSS* | Children and adult* | Not reported | Garg et al. (22) |
| South-east Asia | India | 2014 | Societal** | Public,  Private | Outpatient, Inpatient | Retrospective&  Prospective | Macro-costing | Unclear | Children and adult* | 1,742 | Shepard et al. (23) |
| South-east Asia | India | 2015 | Societal | Private | Inpatient | Prospective | Bottom-up | DF and DHF/DSS* | Children | 757 | Manjunath et al. (24) |
| South-east Asia | India | 2019 | Household | Private | Inpatient | Retrospective | Bottom-up | DF, DHF/DSS | Children, adult | 100 | Panmei et al. (25) |
| South-east Asia | India | 2019 | Societal** | Public, Private | Inpatient | Retrospective | Bottom-up | DF and DHF/DSS* | Children and adult* | 430 | Bajwala et al. (26) |
| South-east Asia | India | 2020 | Household | Public | Outpatient, inpatient | Retrospective | Bottom-up | Unclear | Children and adult* | 83 | Nujum et al. (27) |
| South-east Asia | India | 2021 | Unclear | Public and private* | Inpatient | Prospective | Unclear | Unclear | Children | 560 | Srinivasan et al. (28) |
| South-east Asia | India | 2021 | Household | Public | Inpatient | Retrospective | Bottom-up | Unclear | Adult | 220 | Rafikahmed et al. (29) |
| South-east Asia | India | 2022 | Household | Public, Private | Outpatient and inpatient* | Prospective | Bottom-up | Unclear | Children and adult* | 150 | Kaur et al. (30) |
| South-east Asia | Indonesia | 2019 | Household | Public | Inpatient | Retrospective | Bottom-up | DHF/DSS | Children and adult* | 230 | Supadmi et al. (31) |
| South-east Asia | Indonesia | 2019 | Societal | Private, Public | Outpatient, Inpatient | Retrospective & Prospective | Bottom-up | DF and DHF/DSS* | Children and adult* | 615 | Nadjib et al. (32) |
| South-east Asia | Indonesia | 2020 | Unclear | Public | Outpatient, Inpatient,  Informal care | Prospective | Macro-costing | Unclear | Children and adult* | 67 | Wilastonegoro et al. (33) |
| South-East Asia | Sri Lanka | 2021 | Societal | Public | Inpatient | Prospective | Bottom-up | DF, DHF/DSS | Children | 100 | Sonali Fernando et al. (34) |
| South-East Asia | Sri Lanka | 2021 | Govern-ment | Public | Inpatient | Prospective | Bottom-up | DF, DHF/DSS | Children and adult* | 431 | Sigera et al. (35) |
| South-east Asia | Sri Lanka | 2022 | Societal | Public | Inpatient and ICU* | Prospective | Gross-costing &  Activity-based | DF and DHF/DSS* | Children and adult* | 1,064 | Weerasinghe et al. (36) |
| Americas | Sri Lanka | 2016 | Government | Public | Inpatient,  Intensive care unit | Retrospective | Gross-costing + activity-based | DF, DHF/DSS | Children, adult | 10,017 | Thalagala et al. (37) |
| South-east Asia | Thailand | 1997 | Societal | Public | Inpatient | Prospective | Bottom-up | DHF/DSS | Children, adult | 184 | Okanurak et al. (38) |
| South-east Asia | Thailand | 2005 | Household | Public | Inpatient | Retrospective | Bottom-up | DF and DHF/DSS* | Children and adult* | 238 | Clark et al. (39) |
| South-east Asia | Thailand | 2017 | Household | Public | Inpatient | Prospective | Bottom-up | DF, DHF/DSS | Children, adult | 224 | Tozan et al. (40) |
| Western pacific | Cambodia | 2004 | Household | Public and private | Inpatient and ICU* | Retrospective | Bottom-up | Unclear | Unclear | Unclear | Van Damme et al. (41) |
| Western pacific | Cambodia | 2008 | Household | Public, Private | Inpatient | Prospective | Bottom-up | Unclear | Children | Not reported | Khun et al. (42) |
| Western pacific | Cambodia | 2009 | Household | Public and private* | Outpatient and inpatient* | Prospective | Bottom-up | Unclear | Children | 30 | Huy et al. (43) |
| Western pacific | China | 2017 | Government | Public | Inpatient | Retrospective | Gross-costing | DF and DHF/DSS* | Children, adult | 1,432 | Zhang et al. (44) |
| Western pacific | China | 2022 | Societal | Public | Outpatient and inpatient* | Retrospective | Bottom-up | DF and DHF/DSS* | Children and adult* | 1,040 | Xu et al. (45) |
| Western pacific | China | 2022 | Unclear | Public | Inpatient | Retrospective | Bottom-up | DF | Children | 14 | Wang et al. (46) |
| Western pacific | China | 2023 | Household | Public | Outpatient, Inpatient | Retrospective | Bottom-up | DF and DHF/DSS* | Children and adult* | 340 | Yu et al. (47) |
| Western pacific | Japan | 2020 | Societal** | Public | Outpatient, inpatient | Retrospective | Bottom-up | DF, DHF/DSS | Children and adult* | 1,716 | Kajimoto et al. (48) |
| Western pacific | Malaysia | 2016 | Household | Public | Outpatient and inpatient* | Retrospective | Bottom-up | Unclear | Children and adult* | 355 | Mia et al. (49) |
| Western pacific | Philippines | 2015 | Societal** | Public, Private | Outpatient, Inpatient | Retrospective | Macro-costing | DF and DHF/DSS* | Unclear | 117,065 | Edillo et al. (50) |
| Western pacific | Philippines | 2016 | Societal** | Public and private* | Inpatient | Retrospective | Bottom-up | Unclear | Children, adult | 20,555 | Onuh et al. (51) |
| Western pacific | Viet Nam | 2007 | Household | Public | Inpatient | Unclear | Bottom-up | DHF/DSS | Children | 175 | Harving et al. (52) |
| Western pacific | Viet Nam | 2012 | Household | Public | Inpatient | Retrospective | Bottom-up | Unclear | Children and adult* | 144 | Tam et al. (53) |
| Western pacific | Viet Nam | 2016 | Household | Public | Inpatient | Prospective | Bottom-up | DHF/DSS | Children, adult | 168 | Nhi et al. (54) |
| Western pacific | Viet Nam | 2017 | Unclear | Public | Inpatient | Retrospective | Bottom-up | DF and DHF/DSS* | Children, adult | 263 | Vo et al. (55) |
| Western pacific | Viet Nam | 2017 | Societal | Public | Inpatient | Retrospective | Bottom-up | Unclear | Children, adult | 60 | Pham et al. (56) |
| Western pacific | Viet Nam | 2018 | Household | Public | Outpatient | Retrospective | Bottom-up | DF and DHF/DSS* | Unclear | 123 | Tran et al. (57) |
| Western pacific | Viet Nam | 2019 | Societal | Public | Inpatient and ICU* | Prospective | Bottom-up | DHF/DSS | Adult | 88 | McBride et al. (58) |
| Western pacific | Viet Nam | 2022 | Societal | Public | ICU patient | Retrospective | Bottom-up | Unclear | Adult | 28 | Hung et al. (59) |

Note: * indicates not stratify each sub-group, ** using societal perspective but did not report all cost types.

Abbreviation: WHO, World Health Organization; DF, dengue fever; DHF, dengue haemorrhagic fever; DSS, dengue shock syndrome; ICU, intensive care unit.

**Appendix … Comparison of cost per case between private and public hospitals across countries (2023 USD).**

**Appendix 6 Average reported cost per episode in outpatient private setting across countries (2023 USD).**

|  | Average total cost  (SD) | Average direct medical cost (SD) | Average direct non-medical cost (SD) | Average productivity cost (SD) |
| --- | --- | --- | --- | --- |
| Americas |  |  |  |  |
| Brazil | 252.64 (NA) |  |  | 147.64 (NA) |
| South-east Asia | |  |  |  |
| India |  | 26.58 (NA) |  |  |
| Indonesia | 86.52 (NA) | 33.96 (NA) | 18.98 (NA) | 33.59 (NA) |
| Western pacific | |  |  |  |
| Philippines |  | 165.68 (NA) |  |  |

**Appendix 7 Average reported cost per episode in inpatient private setting across countries (2023 USD)**

|  | Average total cost (SD) | Average direct medical cost (SD) | Average direct non-medical cost(SD) | Average productivity cost (SD) |
| --- | --- | --- | --- | --- |
| Americas |  |  |  |  |
| Brazil |  | 1094.23 (NA) |  | 219.54 (NA) |
| Eastern mediterranean | |  |  |  |
| Pakistan |  | 518.60 (NA) |  |  |
| South-east Asia | |  |  |  |
| Bangladesh | 546.88 (NA) | 341.59 (NA) | 91.24 (NA) | 103.63 (NA) |
| India |  | 449.59 (273.71) |  | 35.94 (33.00) |
| Indonesia | 537.94 (NA) | 359.47 (NA) | 77.17 (NA) | 101.30 (NA) |

**Appendix 8 Result of the linear regression in outpatient public hospitals.**

| Cost type | Number of studies  included | Adjusted R-square | Coefficient | 95% CI Lower  (2.5%) | 95% CI Upper  (97.5%) | Intercept | P-value |
| --- | --- | --- | --- | --- | --- | --- | --- |
| Direct medical cost | 14 | 0.10 | 0.50 | -0.19 | 1.19 | -1.12 | 0.14 |
| Direct non-medical cost | 15 | 0.06 | 0.48 | -0.27 | 1.24 | -2.10 | 0.19 |
| Productivity cost | 15 | 0.39 | 1.04 | 0.32 | 1.75 | -5.50 | 0.008 ** |
| Total cost | 12 | 0.48 | 0.77 | 0.26 | 1.30 | -2.55 | 0.008 ** |

Note: ** indicates a strong statistically significant.

**Appendix 9 Result of the linear regression in inpatient public hospitals.**

| Cost type | Number of studies included | Adjusted R-square | Coefficient | 95% CI Lower  (2.5%) | 95% CI Upper  (97.5%) | Intercept | P-value |
| --- | --- | --- | --- | --- | --- | --- | --- |
| Direct medical cost | 26 | 0.37 | 1.46 | 0.71 | 2.22 | -7.55 | 0.0005 ** |
| Direct non-medical cost | 34 | -0.03 | 0.10 | -0.54 | 0.75 | 2.84 | 0.75 |
| Productivity cost | 33 | 0.48 | 1.03 | 0.65 | 1.42 | -4.62 | 4.81e-06*** |
| Total cost | 21 | 0.45 | 0.96 | 0.48 | 1.44 | -2.57 | 0.0005 ** |

Note: ** indicates a strong statistically significant; *** indicates a very strong statistically significant.

**Appendix 10 Results of the quality assessment of the included studies.**

|  | **Study characteristics** | | | **Methodology and cost analysis** | | | | | | | | | **Result and reporting** | | | | | |
| --- | --- | --- | --- | --- | --- | --- | --- | --- | --- | --- | --- | --- | --- | --- | --- | --- | --- | --- |
| **Assessment** | **Question/ objective** | **Population** | **Perspective** | **Epidemiology approach** | **Costing approach** | **Data collection** | **Identification of resource** | **Measurement of resource** | **Valuation of resource** | **Time horizon** | **Discounting** | **Sensitivity** | **Cost sectors** | **Generalizability** | **Limitations** | **Ethical and  distributional issues** | **Conflict of interest** |  |
| **Yes** | 29% | 61% | 39% | 7% | 21% | 50% | 77% | 100% | 38% | 29% | 0% | 18% | 50% | 84% | 77% | 25% | 75% |  |
| **Partial** | 68% | 36% | 16% | 0% | 0% | 0% | 13% | 0% | 36% | 25% | 5% | 2% | 23% | 7% | 0% | 2% | 0% |  |
| **No** | 0% | 4% | 14% | 93% | 5% | 4% | 11% | 0% | 25% | 38% | 63% | 80% | 27% | 9% | 16% | 71% | 25% |  |
| **Unclear** | 4% | 0% | 30% | 0% | 73% | 46% | 0% | 0% | 2% | 9% | 0% | 0% | 0% | 0% | 7% | 2% | 0% |  |
| **NA** | 0% | 0% | 0% | 0% | 0% | 0% | 0% | 0% | 0% | 0% | 32% | 0% | 0% | 0% | 0% | 0% | 0% |  |

**Appendix 11 Full result of the quality assessment of the included studies.**

| **Study** | **Question/ objective** | **Population** | **Perspective** | **Epidemiology approach** | **Costing approach** | **Data collection** | **Identification of resource** | **Measurement of resource** | **Valuation of resource** | **Time horizon** | **Discounting** | **Sensitivity** | **Cost sectors** | **Generalizability** | **Limitations** | **Ethical and distributional issues** | **Conflict of interest** |
| --- | --- | --- | --- | --- | --- | --- | --- | --- | --- | --- | --- | --- | --- | --- | --- | --- | --- |
| **Yu et al. (47)** | Yes | Yes | Partial | **No** | Unclear | Yes | Yes | Yes | Partial | **No** | NA | No | Yes | Yes | Yes | No | Yes |
| **Sarker et al. (21)** | Yes | Yes | Yes | Yes | Yes | Unclear | Yes | Yes | Partial | Partial | NA | Partial | Yes | Yes | Yes | Yes | Yes |
| **Xu et al. (45)** | Yes | Partial | Partial | No | Unclear | Yes | Yes | Yes | Partial | No | NA | No | Yes | Yes | Yes | No | Yes |
| **Weerasinghe et al. (36)** | Yes | Yes | Yes | No | Yes | Yes | Yes | Yes | Partial | Partial | No | No | Yes | Yes | Yes | No | Yes |
| **Wang et al. (46)** | Partial | Yes | No | Yes | Unclear | Yes | No | Yes | No | Yes | No | No | No | No | Yes | No | Yes |
| **Kaur et al. (30)** | Partial | Partial | Unclear | No | Unclear | Yes | Yes | Yes | Unclear | Unclear | No | No | Yes | Partial | No | No | Yes |
| **Hung et al. (59)** | Yes | Yes | Yes | No | Yes | Unclear | Yes | Yes | Yes | Yes | No | No | Yes | Yes | Yes | Yes | Yes |
| **Abud et al. (10)** | Partial | Partial | Yes | No | Unclear | Yes | Yes | Yes | Yes | Partial | No | No | No | Yes | Yes | No | Yes |
| **Srinivasan et al. (28)** | Partial | Partial | No | No | No | Yes | Yes | Yes | No | Yes | No | No | No | Yes | Unclear | No | Yes |
| **Sonali Fernando et al. (34)** | Partial | Yes | Partial | No | Unclear | Yes | Yes | Yes | Yes | Yes | NA | No | Yes | Yes | Yes | Unclear | No |
| **Sigera et al. (35)** | Partial | Yes | Yes | No | Unclear | Yes | Yes | Yes | Partial | Partial | No | No | Partial | Yes | Yes | No | Yes |
| **Rafikahmed et al. (29)** | Partial | Partial | Unclear | No | Unclear | Yes | Yes | Yes | Partial | Partial | No | No | Yes | Yes | No | No | Yes |
| **Wilastonegoro, et al (33)** | Partial | Partial | Unclear | No | Yes | Yes | Yes | Yes | Yes | Unclear | NA | No | No | Yes | Yes | No | Yes |
| **Nujum et al. (27)** | Yes | Partial | Yes | No | Unclear | Unclear | Yes | Yes | Yes | Partial | NA | No | Yes | Yes | Unclear | No | Yes |
| **Kajimoto et al. (48)** | Partial | Partial | Yes | No | Unclear | Unclear | No | Yes | Partial | Partial | No | Yes | No | Yes | Yes | No | Yes |
| **Akbar et al. (20)** | Partial | Yes | Unclear | No | Yes | Yes | Yes | Yes | Partial | Unclear | No | No | Yes | Yes | Yes | No | Yes |
| **Supadmi et al. (31)** | Partial | Yes | Unclear | No | No | Yes | Yes | Yes | Partial | Unclear | No | No | No | Partial | No | Partial | Yes |
| **Panmei et al. (25)** | Partial | Yes | Unclear | No | Unclear | Unclear | Yes | Yes | Partial | No | No | No | Yes | Yes | Yes | No | Yes |
| **Nadjib et al. (32)** | Partial | Yes | Yes | No | Unclear | Yes | Partial | Yes | Partial | No | No | Yes | Yes | Yes | Yes | No | Yes |
| **McBride et al. (58)** | Partial | Yes | Unclear | No | Unclear | Yes | No | Yes | Partial | No | No | No | No | Yes | Yes | Yes | Yes |
| **Lee et al. (60)** | Yes | Yes | Yes | No | Unclear | Unclear | Yes | Yes | Partial | No | No | No | Partial | Yes | Yes | Yes | Yes |
| **Jamil et al. (19)** | Yes | Partial | Yes | No | Unclear | Unclear | Yes | Yes | Partial | Yes | No | No | Yes | Partial | No | No | Yes |
| **Bajwala et al. (26)** | Partial | Yes | Partial | Yes | Unclear | Yes | Yes | Yes | Yes | Yes | No | No | Yes | Yes | Yes | Yes | Yes |
| **Tran et al. (57)** | Partial | Yes | Unclear | No | Unclear | Yes | Yes | Yes | No | No | NA | No | Yes | Yes | Yes | No | Yes |
| **Godói et al. (61)** | Yes | Yes | Yes | No | No | Unclear | Partial | Yes | Partial | Partial | No | No | No | Yes | Yes | No | Yes |
| **Zhang et al. (44)** | Partial | Yes | Yes | No | Unclear | Unclear | Yes | Yes | Yes | Yes | No | No | Yes | Yes | Yes | No | Yes |
| **Vo et al. (55)** | Partial | Yes | No | No | Unclear | Yes | Yes | Yes | Partial | Partial | No | No | Yes | Yes | Unclear | No | No |
| **Tozan et al. (40)** | Yes | Yes | Yes | No | Unclear | Yes | Yes | Yes | Partial | Unclear | No | No | Partial | Yes | Yes | No | Yes |
| **Pham et al. (56)** | Yes | Partial | Partial | Yes | Unclear | Yes | Yes | Yes | Partial | Partial | No | Yes | Yes | Yes | Yes | Yes | Yes |
| **Legorreta-Soberanis et al. (14)** | Yes | Yes | Partial | No | Unclear | Unclear | Yes | Yes | Yes | Partial | No | No | No | No | Yes | Yes | Yes |
| **Lee et al. (60)** | Partial | Partial | Unclear | No | Unclear | Unclear | Yes | Yes | Yes | Yes | No | No | Partial | Yes | Yes | No | Yes |
| **Zubieta-Zavala et al. (13)** | Partial | Partial | Yes | No | Yes | Unclear | Yes | Yes | Yes | No | NA | No | Yes | Yes | Yes | No | Yes |
| **Uhart et al. (12)** | Partial | No | Partial | No | Unclear | Unclear | Yes | Yes | Partial | Yes | No | No | No | Yes | Yes | No | Yes |
| **Thalagala et al. (37)** | Yes | Partial | Yes | No | Yes | Yes | Yes | Yes | Yes | Yes | NA | No | Yes | Yes | Yes | No | Yes |
| **Onuh et al. (51)** | Partial | Partial | Unclear | No | Unclear | Unclear | Partial | Yes | Yes | No | No | No | Partial | Yes | Yes | No | Yes |
| **Nhi et al. (54)** | Partial | Yes | Unclear | No | Unclear | Unclear | Yes | Yes | Yes | Yes | No | No | Yes | Yes | Yes | No | Yes |
| **Mia et al. (49)** | Partial | Partial | Yes | No | Unclear | Yes | Yes | Yes | Yes | Yes | No | No | Yes | Yes | Yes | No | No |
| **Salmon-Mulanovich et al. (16)** | Yes | Yes | Yes | No | Unclear | Unclear | Yes | Yes | Yes | No | No | No | Partial | Yes | Yes | Yes | No |
| **Rafique et al. (18)** | Partial | Yes | Yes | No | Unclear | Unclear | Yes | Yes | No | Yes | No | No | Partial | Yes | Yes | No | No |
| **Martelli et al. (8)** | Yes | Yes | Yes | No | Yes | Yes | Yes | Yes | Yes | Partial | No | Yes | Partial | Yes | Yes | No | Yes |
| **Manjunath et al. (24)** | Partial | Yes | Unclear | No | Unclear | Unclear | No | Yes | No | No | NA | No | Partial | Partial | No | No | Yes |
| **Castro Rodriguez et al. (11)** | Partial | Yes | Yes | No | Unclear | Unclear | Yes | Yes | Yes | No | Partial | Yes | Yes | Yes | Yes | No | No |
| **Edillo et al. (50)** | Partial | Partial | No | No | Unclear | Unclear | Yes | Yes | Partial | No | No | Yes | Yes | Yes | Yes | No | No |
| **Vieira et al. (7)** | Partial | Yes | Partial | No | Yes | Yes | Yes | Yes | Yes | No | NA | No | Yes | Yes | Yes | Yes | Yes |
| **Shepard et al. (23)** | Partial | Partial | No | No | Yes | Yes | Yes | Yes | Yes | Yes | No | Yes | Partial | Yes | Yes | No | No |
| **Tam et al. (53)** | Partial | Partial | Unclear | No | Unclear | Unclear | No | Yes | No | Partial | No | No | Yes | Yes | No | Yes | Yes |
| **Suaya et al. (6)** | Partial | Yes | Yes | No | Yes | Yes | Yes | Yes | Yes | Yes | Partial | No | Partial | Yes | Yes | No | Yes |
| **Riaz et al. (17)** | Partial | Yes | No | No | Unclear | Unclear | Partial | Yes | No | No | NA | No | No | No | No | No | No |
| **Huy et al. (43)** | Yes | Partial | Unclear | No | Unclear | Yes | Yes | Yes | Yes | No | NA | No | Partial | Yes | Yes | Yes | Yes |
| **Khun et al. (42)** | Unclear | Yes | Unclear | No | Unclear | Unclear | Yes | Yes | No | No | No | No | No | Yes | No | Yes | Yes |
| **Garga et al. (22)** | Partial | Yes | No | No | Unclear | No | Yes | Yes | No | No | NA | Yes | No | Yes | Yes | No | Yes |
| **Armien et al. (15)** | Partial | Yes | Yes | No | Yes | Yes | Partial | Yes | No | No | Partial | Yes | Partial | No | Yes | No | No |
| **Harving et al. (52)** | Partial | Yes | No | No | Unclear | No | Yes | Yes | No | No | NA | No | Yes | Yes | Yes | No | No |
| **Clark et al. (39)** | Partial | Partial | Unclear | No | Unclear | Unclear | Partial | Yes | No | Yes | NA | Yes | No | Yes | No | No | No |
| **Van Damme et al. (41)** | Unclear | No | Unclear | No | Unclear | Unclear | No | Yes | No | No | NA | No | No | Yes | Yes | Yes | No |
| **Okanurak et al. (38)** | Partial | Yes | Unclear | No | Unclear | Unclear | Partial | Yes | No | Partial | NA | No | Yes | No | Unclear | Yes | No |

**Appendix 12 Comparison of the linear regressions between this study and Shepard et al. (62) (2023 USD): Panel A presents direct cost comparisons, combining projected direct medical and non-medical costs from this study (hence the non-linear trend). Panel B presents productivity cost comparisons.**

1. **Direct cost comparisons** **(B) Productivity cost comparisons**


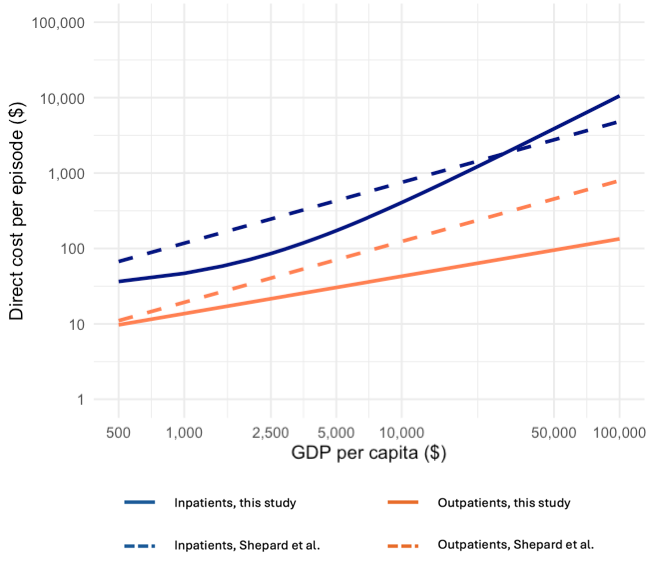


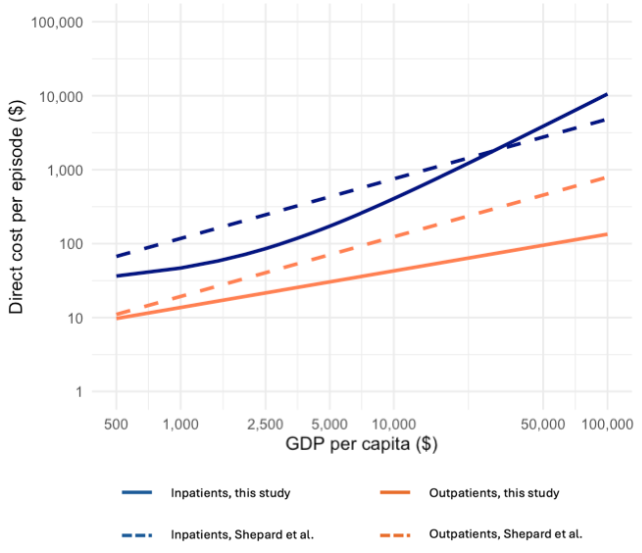

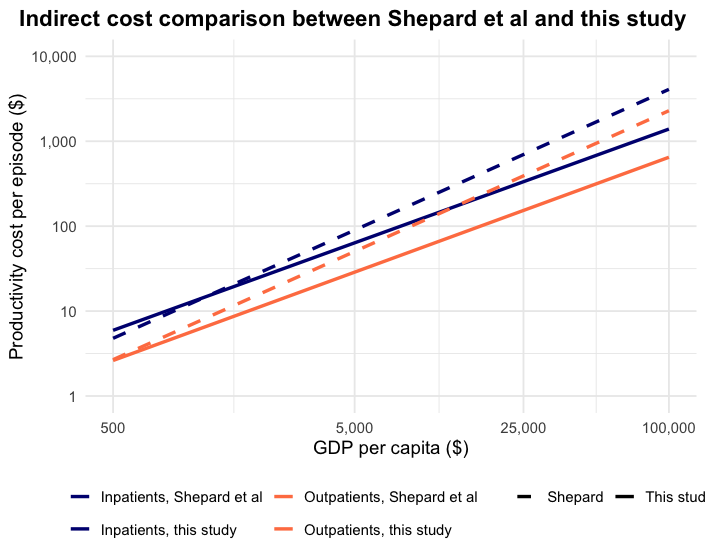


**References**

1. Page MJ, McKenzie JE, Bossuyt PM, Boutron I, Hoffmann TC, Mulrow CD, et al. The PRISMA 2020 statement: an updated guideline for reporting systematic reviews. BMJ. 2021;372:n71.

2. Turner HC, Lauer JA, Tran BX, Teerawattananon Y, Jit M. Adjusting for inflation and currency changes within health economic studies. Value in Health. 2019;22(9):1026-32.

3. International Monetary Fund. GDP per capita, current prices 2023 [cited 2024 17 August]. Available from: <https://www.imf.org/external/datamapper/NGDPDPC@WEO/OEMDC/ADVEC/WEOWORLD>.

4. Lee J-S, Mogasale V, Lim JK, Ly S, Lee KS, Sorn S, et al. A multi-country study of the economic burden of dengue fever based on patient-specific field surveys in Burkina Faso, Kenya, and Cambodia. PLoS neglected tropical diseases. 2019;13(2):e0007164.

5. Lee J-S, Mogasale V, Lim JK, Carabali M, Lee K-S, Sirivichayakul C, et al. A multi-country study of the economic burden of dengue fever: Vietnam, Thailand, and Colombia. PLoS neglected tropical diseases. 2017;11(10):e0006037.

6. Suaya JA, Shepard DS, Siqueira JB, Martelli CT, Lum LCS, Tan LH, et al. Cost of dengue cases in eight countries in the Americas and Asia: a prospective study. The American journal of tropical medicine and hygiene. 2009;80(5):846-55.

7. Vieira Machado AA, Estevan AO, Sales A, Brabes KCdS, Croda J, Negrao FJ. Direct costs of dengue hospitalization in Brazil: public and private health care systems and use of WHO guidelines. PLoS neglected tropical diseases. 2014;8(9):e3104.

8. Martelli CMT, Siqueira JB, Jr., Parente MPPD, Zara ALdSA, Oliveira CS, Braga C, et al. Economic Impact of Dengue: Multicenter Study across Four Brazilian Regions. PLoS neglected tropical diseases. 2015;9(9):e0004042.

9. Godói IP, Da Silva LVD, Sarker AR, Megiddo I, Morton A, Godman B, et al. Economic and epidemiological impact of dengue illness over 16 years from a public health system perspective in Brazil to inform future health policies including the adoption of a dengue vaccine. Expert Review of Vaccines. 2018;17(12):1123-33.

10. Abud DA, Santos CY, Neto AAL, Senra JT, Tuboi S. Real world data study of prevalence and direct costs related to dengue management in Brazil's private healthcare from 2015 to 2020. Braz J Infect Dis. 2022;26(6):102718.

11. Castro Rodriguez R, Galera-Gelvez K, Lopez Yescas JG, Rueda-Gallardo JA. Costs of dengue to the health system and individuals in Colombia from 2010 to 2012. The American journal of tropical medicine and hygiene. 2015;92(4):709-14.

12. Uhart M, Blein C, L'Azou M, Thomas L, Durand L. Costs of dengue in three French territories of the Americas: an analysis of the hospital medical information system (PMSI) database. The European journal of health economics : HEPAC : health economics in prevention and care. 2016;17(4):497-503.

13. Zubieta-Zavala A, Salinas-Escudero G, Ramirez-Chavez A, Garcia-Valladares L, Lopez-Cervantes M, Lopez Yescas JG, et al. Calculation of the Average Cost per Case of Dengue Fever in Mexico Using a Micro-Costing Approach. PLoS neglected tropical diseases. 2016;10(8):e0004897.

14. Legorreta-Soberanis J, Paredes-Solis S, Morales-Perez A, Nava-Aguilera E, Serrano-de Los Santos FR, Dimas-Garcia DL, et al. Household costs of dengue illness: secondary outcomes from a randomised controlled trial of dengue prevention in Guerrero state, Mexico. BMC public health. 2017;17(Suppl 1):411.

15. Armien B, Suaya JA, Quiroz E, Sah BK, Bayard V, Marchena L, et al. Clinical characteristics and national economic cost of the 2005 dengue epidemic in Panama. The American journal of tropical medicine and hygiene. 2008;79(3):364-71.

16. Salmon-Mulanovich G, Blazes DL, Lescano AG, Bausch DG, Montgomery JM, Pan WK. Economic Burden of Dengue Virus Infection at the Household Level Among Residents of Puerto Maldonado, Peru. The American journal of tropical medicine and hygiene. 2015;93(4):684-90.

17. Riaz MM, Mumtaz K, Khan MS, Patel J, Tariq M, Hilal H, et al. Outbreak of dengue fever in Karachi 2006: a clinical perspective. JPMA The Journal of the Pakistan Medical Association. 2009;59(6):339-44.

18. Rafique I, Nadeem Saqib MA, Munir MA, Qureshi H, Siddiqui S, Habibullah S, et al. Economic burden of dengue in four major cities of Pakistan during 2011. JPMA The Journal of the Pakistan Medical Association. 2015;65(3):256-9.

19. Jamil F, Asif A, Farooq F. A RESEARCH STUDY TO ASSESS THE PATIENT'S EXPENDITURE SUFFERING FROM DENGUE FEVER WITH RESPECT TO COUNTRY'S LOSS AND PATIENT'S CURE. INDO AMERICAN JOURNAL OF PHARMACEUTICAL SCIENCES. 2019;6(2).

20. Akbar NA, Assiri AM, Shabouni OI, Alwafi OM, Al-Raddadi R, H Alzahrani M, et al. The economic burden of dengue fever in the Kingdom of Saudi Arabia. PLoS neglected tropical diseases. 2020;14(11):e0008847.

21. Sarker AR, Paul S, Zohara F, Hossain Z, Zabeen I, Chowdhury SMZI, et al. Economic burden of dengue in urban Bangladesh: A societal perspective. PLoS neglected tropical diseases. 2023;17(12):e0011820.

22. Garg P, Nagpal J, Khairnar P, Seneviratne SL. Economic burden of dengue infections in India. Transactions of the Royal Society of Tropical Medicine and Hygiene. 2008;102(6):570-7.

23. Shepard DS, Halasa YA, Tyagi BK, Adhish SV, Nandan D, Karthiga KS, et al. Economic and disease burden of dengue illness in India. The American journal of tropical medicine and hygiene. 2014;91(6):1235-42.

24. Manjunath MN, Nair CC, Sharanya R. A study on clinical features and cost incurred by dengue syndrome patients admitted to tertiary care hospital. British Journal of Medical Practitioners. 2015;8(2):a811.

25. Panmei K, Joseph AK, Rose W, Abraham OC, Mathuram AJ, Kumar S, et al. Direct cost of illness for dengue in hospitalized children and adults at a referral hospital in India. International Journal of Infectious Diseases. 2019;84(Supplement):S64-S7.

26. Bajwala VR, John D, Rajasekar TD, Murhekar MV. Severity and costs associated with hospitalization for dengue in public and private hospitals of Surat city, Gujarat, India, 2017-2018. Transactions of the Royal Society of Tropical Medicine and Hygiene. 2019;113(11):661-9.

27. Nujum ZT, Beegum MS, Meenakshy V, Vijayakumar K. Cost analysis of dengue from a State in south India. The Indian journal of medical research. 2020;152(5):490-7.

28. Srinivasan M, Sindhu KN, Nag A, Karthikeyan AS, Ramasamy RK, Murugesan M, et al. Hospitalization Rates and Direct Medical Costs for Fever in a Pediatric Cohort in South India. The Journal of infectious diseases. 2021;224(Supple 5):S548-S57.

29. Rafikahmed SR, Mateti UV, Subramanya C, Shetty S, Sunny A, Madhusoodanan A. Assessment of direct medical cost using cost of illness analysis in patients with dengue fever - Retrospective study. Clinical Epidemiology and Global Health. 2021;12((Rafikahmed, Mateti, Sunny, Madhusoodanan) Nitte (Deemed to be University), NGSM Institute of Pharmaceutical Sciences, Department of Pharmacy Practice, Deralakatte, Mangaluru, Karnataka, India(Subramanya) Nitte (Deemed to be University), KS Hegde Medical):100842.

30. Kaur J, Yadav CP, Chauhan NM, Baharia RK. Economic burden estimation associated with dengue and chikungunya in Gujarat, India. Journal of family medicine and primary care. 2022;11(9):5393-403.

31. Supadmi W, Izzah QN, Suwantika AA, Perwitasari DA, Abdulah R. Cost of Illness Study of Patients with Dengue Hemorrhagic Fever at One of the Private Hospitals in Yogyakarta. Journal of pharmacy & bioallied sciences. 2019;11(Suppl 4):S587-S93.

32. Nadjib M, Setiawan E, Putri S, Nealon J, Beucher S, Hadinegoro SR, et al. Economic burden of dengue in Indonesia. PLoS neglected tropical diseases. 2019;13(1):e0007038.

33. Wilastonegoro NN, Kharisma DD, Laksono IS, Halasa-Rappel YA, Brady OJ, Shepard DS. Cost of Dengue Illness in Indonesia across Hospital, Ambulatory, and not Medically Attended Settings. The American journal of tropical medicine and hygiene. 2020;103(5):2029-39.

34. Sonali Fernando E, Headley TY, Tissera H, Wilder-Smith A, De Silva A, Tozan Y. Household and Hospitalization Costs of Pediatric Dengue Illness in Colombo, Sri Lanka. The American journal of tropical medicine and hygiene. 2021;105(1):110-6.

35. Sigera C, Rodrigo C, de Silva NL, Weeratunga P, Fernando D, Rajapakse S. Direct costs of managing in-ward dengue patients in Sri Lanka: A prospective study. PLoS ONE. 2021;16(10 October):e0258388.

36. Weerasinghe NP, Bodinayake CK, Wijayaratne WMDGB, Devasiri IV, Dahanayake NJ, Kurukulasooriya MRP, et al. Direct and indirect costs for hospitalized patients with dengue in Southern Sri Lanka. BMC health services research. 2022;22(1):657.

37. Thalagala N, Tissera H, Palihawadana P, Amarasinghe A, Ambagahawita A, Wilder-Smith A, et al. Costs of Dengue Control Activities and Hospitalizations in the Public Health Sector during an Epidemic Year in Urban Sri Lanka. PLoS neglected tropical diseases. 2016;10(2):e0004466.

38. Okanurak K, Sornmani S, Indaratna K. The cost of dengue hemorrhagic fever in Thailand. The Southeast Asian journal of tropical medicine and public health. 1997;28(4):711-7.

39. Clark DV, Mammen MP, Jr., Nisalak A, Puthimethee V, Endy TP. Economic impact of dengue fever/dengue hemorrhagic fever in Thailand at the family and population levels. The American journal of tropical medicine and hygiene. 2005;72(6):786-91.

40. Tozan Y, Ratanawong P, Sewe MO, Wilder-Smith A, Kittayapong P. Household costs of hospitalized dengue illness in semi-rural Thailand. PLoS neglected tropical diseases. 2017;11(9):e0005961.

41. Van Damme W, Van Leemput L, Por I, Hardeman W, Meessen B. Out-of-pocket health expenditure and debt in poor households: evidence from Cambodia. Tropical medicine & international health : TM & IH. 2004;9(2):273-80.

42. Khun S, Manderson L. Poverty, user fees and ability to pay for health care for children with suspected dengue in rural Cambodia. International Journal for Equity in Health. 2008;7((Khun) National Centre for Health Promotion, Ministry of Health, Phnom Penh, Cambodia(Manderson) School of Psychology, Psychiatry and Psychological Medicine, Faculty of Medicine, Nursing and Health Sciences, Monash University, Clayton, VIC, Australia):10.

43. Huy R, Wichmann O, Beatty M, Ngan C, Duong S, Margolis HS, et al. Cost of dengue and other febrile illnesses to households in rural Cambodia: a prospective community-based case-control study. BMC public health. 2009;9(100968562):155.

44. Zhang JH, Yuan J, Wang T. Direct cost of dengue hospitalization in Zhongshan, China: Associations with demographics, virus types and hospital accreditation. PLoS neglected tropical diseases. 2017;11(8):e0005784.

45. Xu M, Chang N, Tu T, Sun J, Jiang J, Xia Y, et al. Economic burden of dengue fever in China: A retrospective research study. PLoS neglected tropical diseases. 2022;16(5):e0010360.

46. Wang R, Wang X, Zhang L, Feng G, Liu M, Zeng Y, et al. The epidemiology and disease burden of children hospitalized for viral infections within the family Flaviviridae in China: A national cross-sectional study. PLoS neglected tropical diseases. 2022;16(7):e0010562.

47. Yu Y, Liu Y, Ling F, Sun J, Jiang J. Epidemiological Characteristics and Economic Burden of Dengue in Zhejiang Province, China. Viruses. 2023;15(8).

48. Kajimoto Y, Kitajima T. Patient and National Economic Burden of Dengue in Japan: Results from Japanese National Claims Database. The American journal of tropical medicine and hygiene. 2020;102(6):1237-43.

49. Mia MS, Begum RA, Er AC, Pereira JJ. ASSESSING THE COST BURDEN OF DENGUE INFECTION TO HOUSEHOLDS IN SEREMBAN, MALAYSIA. The Southeast Asian journal of tropical medicine and public health. 2016;47(6):1167-76.

50. Edillo FE, Halasa YA, Largo FM, Erasmo JNV, Amoin NB, Alera MTP, et al. Economic cost and burden of dengue in the Philippines. The American journal of tropical medicine and hygiene. 2015;92(2):360-6.

51. Onuh W, Cabanacan-Salibay C, Manaig P. Economic costs and burden of dengue disease in cavite province, Philippines. Scientia Medica. 2016;26(2):1-11.

52. Harving ML, Ronsholt FF. The economic impact of dengue hemorrhagic fever on family level in Southern Vietnam. Danish medical bulletin. 2007;54(2):170-2.

53. Tam PT, Dat NT, Huu LM, Thi XCP, Duc HM, Tu TC, et al. High household economic burden caused by hospitalization of patients with severe dengue fever cases in Can Tho province, Vietnam. The American journal of tropical medicine and hygiene. 2012;87(3):554-8.

54. Nhi TNY, Trung VQ. The economic value of informal care for dengue patients in vietnam. International Journal of Research in Ayurveda and Pharmacy. 2016;7(6):101-6.

55. Vo NTT, Phan TND, Vo TQ. Direct Medical Costs of Dengue Fever in Vietnam: A Retrospective Study in a Tertiary Hospital. The Malaysian journal of medical sciences : MJMS. 2017;24(3):66-72.

56. Pham LD, Phung NHT, Le NTD, Vo TQ. Economic report on the cost of dengue fever in Vietnam: case of a provincial hospital. ClinicoEconomics and outcomes research : CEOR. 2017;9(101560564):1-8.

57. Tran BX, Thu Vu G, Hoang Nguyen L, Tuan Le Nguyen A, Thanh Tran T, Thanh Nguyen B, et al. Cost-of-Illness and the Health-Related Quality of Life of Patients in the Dengue Fever Outbreak in Hanoi in 2017. International journal of environmental research and public health. 2018;15(6).

58. McBride A, Thuy Duong B, Chau Nguyen VV, Thwaites CL, Turner HC, Hao Nguyen V. Catastrophic health care expenditure due to septic shock and dengue shock in Vietnam. Transactions of the Royal Society of Tropical Medicine and Hygiene. 2019;113(10):649-51.

59. Hung TM, Van Hao N, Yen LM, McBride A, Dat VQ, van Doorn HR, et al. Direct Medical Costs of Tetanus, Dengue, and Sepsis Patients in an Intensive Care Unit in Vietnam. Frontiers in public health. 2022;10(101616579):893200.

60. Lee JS, Mogasale V, Lim JK, Ly S, Lee KS, Sorn S, et al. A multi-country study of the economic burden of dengue fever based on patient-specific field surveys in Burkina Faso, Kenya, and Cambodia. PLoS Negl Trop Dis. 2019;13(2):e0007164.

61. Godoi IP, Da Silva LVD, Sarker AR, Megiddo I, Morton A, Godman B, et al. Economic and epidemiological impact of dengue illness over 16 years from a public health system perspective in Brazil to inform future health policies including the adoption of a dengue vaccine. Expert review of vaccines. 2018;17(12):1123-33.

62. Shepard DS, Undurraga EA, Halasa YA, Stanaway JD. The global economic burden of dengue: a systematic analysis. The Lancet Infectious Diseases. 2016;16(8):935-41.
